# Effect of Master Alloy Based on Al and Si with Ti and B on Mechanical Properties of AlSi9 Alloy

**DOI:** 10.3390/ma19020431

**Published:** 2026-01-22

**Authors:** Tomasz Lipiński

**Affiliations:** Faculty of Technical Sciences, University of Warmia and Mazury in Olsztyn, 10-719 Olsztyn, Poland; tomekl@uwm.edu.pl

**Keywords:** Al-Si alloys, silumin, modification, mechanical properties

## Abstract

Hypoeutectic aluminum–silicon casting alloys in their unmodified state have a coarse-grained eutectic (α + β), which results in poor mechanical properties and brittleness. Microstructure refinement and improved mechanical properties are possible, among other things, by introducing various elements and chemical compounds. The literature presents numerous studies on the modification of hypoeutectic silumins, but there are no results confirming the effectiveness of the interaction of a master alloy containing titanium and boron with its main component, which may be aluminum, aluminum with silicon, or aluminum with silicon and magnesium. This paper presents the results of microstructure refinement using titanium or boron introduced into the Al, AlSi7, and AlSi7Mg master alloys. The introduction of titanium and boron into the aluminum-based master alloy resulted in microstructure refinement and improved mechanical properties. The results indicate that the most favorable results were obtained when titanium and boron were introduced into the AlSi7 master alloy. The addition of magnesium to the master alloy AlSi7 resulted in less effective microstructure refinement of the AlSi9 silumin, which resulted in lower mechanical properties than those obtained for the master alloy without Mg.

## 1. Introduction

Aluminum–silicon alloys have been known and successfully used for many years. Hypoeutectic alloys produced by metallurgical processes and not subjected to quenching and tempering have a thick eutectic composed of a solid solution of silicon in aluminum (α) and a solid solution of aluminum in silicon (β) against the background of the primary α phase [1,2,3,4], which is a consequence of the crystallization process [5,6,7]. They have high mechanical properties [8,9,10,11] and good corrosion resistance [12]. Silumins often contain impurities that deteriorate their mechanical properties and must be eliminated from the alloy, e.g., iron compounds [13]. For applications in load-bearing components, it is necessary to improve their low mechanical properties [14,15,16]. This process can be achieved by several methods [17,18,19,20]. Known technological methods include directional crystallization [21,22,23], focused heat treatment [24,25,26,27,28], electron-plasma alloying [29], heat treatment [30,31,32,33,34], or as a result of their refining process [35,36]. Results confirming the influence of equal channel angular pressing (ECAP) and multi-axial compression deformation (MAC) on the grain refinement of Al-Si alloys [37] have also been presented. The possibility of refining the microstructure of hypoeutectic Al-Si alloys by introducing a modifier called homogeneous [38,39] produced by rapid cooling of the Al-Si alloy and based on the treated alloy [40,41] has been demonstrated. This is an interesting method because it does not introduce chemical elements other than those naturally present in the treated alloy into the treated alloy. Methods for enhancing the properties of silumins with chemical elements and compounds have gained greater popularity. The presented research results confirm good results in modifying the microstructure and thus improving the mechanical properties of hypoeutectic and near-eutectic silumins using sodium [42] or, more commonly, strontium [43,44,45,46], and complex mixtures containing sodium or strontium [47,48,49,50,51,52], or strontium and other technological factors, such as rotating magnetic fields [53]. Although developed many years ago, this method is still used today. Research results using modifiers containing sodium or strontium produced in various technological processes are known [54,55,56]. Good results were obtained by introducing silumin-enhancing components into the exothermic mixture [57,58,59].

There are also studies confirming the possibility of using other elements to modify the microstructure and properties of hypoeutectic silumin, such as yttrium [60], yttrium oxide [61], lithium [62], calcium [63], antimony [64], copper [65], or complex mixtures, e.g., with molybdenum, zirconium, and copper [66], or a Cl-based modifier [67]. Favorable properties have been obtained using modifiers composed of several chemical elements [68,69,70,71]. Good microstructure refinement effects on hypoeutectic and peri-eutectic silumins can be achieved by treatment with titanium [72,73,74,75], titanium and boron [76,77,78,79,80], or alloys containing these components [81,82,83,84]. The authors [72] point to the nucleating properties of AlB2 under the activating action of dissolved Si, which results in grain refinement of the α phase and thus in microstructure refinement that may influence the mechanical properties of silumin. In [74], it was shown that microalloying Al-12.5wt.%Si-0.5wt.%Mg with 0.1wt.% Ti resulted in about 50% higher tensile strength compared to the starting alloy. Ti concentrations exceeding 0.1% in A356 and A357 alloys caused grain size growth in Al-Si alloys, resulting in a decrease in the mechanical properties of silumin [74]. However, the Ti interaction weakens with the number of silumin remelts [73]. Chemical compounds containing these elements are also used [85,86,87,88]. Titanium and boron are often introduced in master alloys in combination with aluminum, titanium [89,90] or other elements [91,92,93,94] or master alloys with the chemical composition of the treated alloy with titanium or boron [95,96]. It was noted that the master alloy Al-3V-3B refines the α-phase grains several times similarly to AlB2 [90]. Similar results were obtained for Al-Ti-Nb-B with the proportions Ti:Nb = 1:4 and M:B = 8:1 [91]. Interesting research results on hypoeutectic silumin treated with boron and strontium [97] have also been presented. Improved properties of silumins have also been demonstrated after treatment with a multi-component mixture of Al-5Ti-0.25C-0.25B [98] and Al-Ti-Nb-B [99]. Numerous studies present the results of improving the microstructure and mechanical properties of Al-Si alloys with titanium and boron [72,73,74,75,76,77,78,79,80,81,82,83,84,85,86,87,88,89,90,91,92,93,94,95,96]. Many studies demonstrate a more intense effect of master alloy than single modifying components [58,89,95,98]. The well-known Al5Ti1B master alloy is often used for treatment [30,46,48,73,81,82,87,92,95,97]. Its use has been reported to refine the microstructure and simultaneously improve the mechanical properties of hypoeutectic silumin. Master alloy Al5Ti1B is readily used in many applications due to its wide availability and the lack of manufacturing requirements. Studies [100,101] have shown that these proportions are not optimal for ensuring high-silumin properties. The optimal Ti:B ratio was considered to be 2:1 [102]. Additionally, it has been found that introducing other chemical elements into the master alloy containing titanium and boron further enhances the properties of Al-Si alloys [100,101,102]. Many authors point to the beneficial effect of titanium and boron introduced with Al [79,80,81,82,87,88,89,90,91,92,93,94,95,96,97,98,99,100,101,102]. Analyzing the results of research on improving the microstructure of hypoeutectic and peri-eutectic silumins with titanium and boron, it was found that better results are obtained after introducing these additions in a master alloy containing the main alloy components, which are aluminum and silicon, than in the case of pure chemical elements. It was also shown that a master alloy with the composition of the processed alloy produced by rapid cooling can also affect the eutectic grain size (α + β) of hypo- and peri-eutectic silumins [38,39]. Despite the large number of studies describing the effect of titanium and boron-based master alloys on the microstructure and mechanical properties of hypoeutectic silumins, there are still no results confirming the effectiveness of the interaction of a titanium and boron-based master alloy with its main component, which may be aluminum, aluminum with silicon, or aluminum with silicon and magnesium. This study compares the effects of the main component of the master alloy, aluminum with silicon, or aluminum with silicon and magnesium, and the addition of titanium and boron on the microstructure and mechanical properties of hypoeutectic silumin. A research hypothesis was also put forward: the effectiveness of the addition of titanium and boron on the mechanical properties of hypoeutectic silumin will depend on the chemical composition of the master alloy in which it is introduced.

## 2. Materials and Methods

The tests were carried out on the EN 1706 AC-AlSi9 (ENAC-44400) [103] alloy. The chemical composition tested silumin is shown in Table 1.

This study used an industrial alloy collected from the foundry before the inoculant was introduced. Each melt was conducted using approximately 800 g of silumin. The AlSi9 silumin intended for testing, with the chemical composition shown in Table 1, was melted in a 2.5 dm^3^ ceramic crucible made of Al_2_O_3_. The melting process was conducted in an electric furnace. After preparation, the master alloy was introduced into a crucible with liquid AlSi9 silumin at 700 °C. After the inoculant was added, the alloy was held in the liquid state for approximately 10 min.

To prepare the master alloy, titanium powder with a purity of >98.5% Ti, boron powder with a purity of >95% B, magnesium powder with a purity of >95% Mg, aluminum pellets with a purity of >98% Al, and silicon pellets with a purity of >95% Si were used. A master alloy composed of Al + 7% Si + Mg + (Ti and B) and Al + 7% Si + (Ti and B) was prepared by melting appropriate amounts of Al and Si in a ceramic crucible. The resulting alloy was cooled to room temperature, forming ingots weighing approximately 50 g. The master alloy component was produced in a similar manner to the previous method, obtaining an Al-7% Si alloy. Mg was added to the resulting alloy while still in a liquid state. The resulting Al-Si-Mg alloy was cooled to ambient temperature. The master alloy was prepared by adding Ti and B to the appropriate amount of previously prepared Al-Si or Al-Si-Mg alloy, according to the test plan. The mass of added titanium and boron was calculated based on the mass of the corresponding Al-Si or Al-Si-Mg alloy and the mass of the Al-9% Si alloy it was intended to process. When selecting the proportions of titanium and boron, care was taken to ensure the appropriate proportions of each of the introduced components in the treated alloy, in accordance with the research plan. The master alloy was produced in an electric furnace at 750 °C. The proportions of modifying components were determined based on preliminary tests [104] at the following levels: 0.04% Ti, 0.02% B.

The total weight of the additives in the master alloy ranged around 10% Al-Si or Al-Si-Mg. The liquid master alloy was solidified by pouring it onto a metal drum heated to approximately 100 °C. The mixture was then mechanically crushed and screened to a fraction of 0.40–0.63 mm. The master alloy with additives was kept in the liquid state for approximately 10 min before casting. After processing, the silumin was siphon cast into a sand mold. Each casting was used to produce three vertically cast cylindrical specimens, 10 mm in diameter and 130 mm long. After the casting solidified, the gating system was cut off. Two independent melts were performed for each plan point. Static tensile test specimens were made from each casting with the following dimensions: test piece diameter d_0_ = 6 mm, original gauge length (L_0_ = 5 d_0_) = 30 mm, length of gripped ends h = 30 mm, diameter of gripped ends d1 = 9 mm, and total length of test piece Lt = 102 mm. Hardness (H) was tested using the Brinell method (HB) on one of the heads of each strength specimen. For this purpose, the side surface was ground to create a test surface. Before the tensile test, Brinell hardness was measured according to ISO 6506-1:2014 [104] using a 2.5 mm diameter steel ball at a load of 306.5 N and a load time of 20 s using the HPO 250 Brinell/Vickers tester (VEB Werkstoffprufmaschinenkombinat “Fritz Heckert”, Leipzig, Germany). Three measurements were taken on each sample head. The static tensile test was performed on a ZD10 (VEB Werkstoffprufmaschinenkombinat “Fritz Heckert”, Leipzig, Germany) machine according to EN ISO 6892-1:2016 [105]. Tensile strength (Rm) and relative elongation (A) were determined. Strength was tested on three samples from each casting. It was assumed that if the discrepancy in the results between the average values obtained for individual castings at the same point in the test plan differed by 10% or more, a root cause analysis would be performed, followed by another casting at that point in the test plan, and all mechanical tests would be repeated (due to the laboratory conditions of the melts, this was not necessary). To faithfully reproduce the microstructure of the tested silumin after treatment with individual modifiers, it was decided to conduct metallographic studies directly on sections from the samples on which mechanical tests were conducted. For samples from each melt representing average mechanical properties, sections were taken from a location that was slightly deformed due to the static tensile test. Metallographic sections were made on the transverse planes of the sections lying between the original gauge length and the beginning of the sample transition radius at the length of the gripped ends. The metallographic sections were etched with Mi8Al. Microscopic observation was performed using an Olympus IX70 optical microscope (Olympus, Shinjuku, Japan). Fractures were also observed using a Jeol JSM-7100 SEM microscope (JEOL Ltd., Tokyo, Japan). Phase analysis was performed using a Philips X’Pert PW 1710 diffractometer (PANalytical, Almelo, The Netherlands). In both cases, Co-Kα radiation (λ = 1.78897 Å) was used. Microstructure analysis was conducted, and stereological parameters were determined using an Olympus IX 70 S8F2 (Olympus, Shinjuku, Japan) optical microscope with DP Soft ver. 3.2 software. The following were determined:

- tβ—average thickness of the β phase in the eutectic, μm.
- lβ—average length of the β phase in the eutectic, μm.
- tα—average thickness of the α phase dendrite arms, μm.
- aβ—average product of the average thickness of the β phase in the eutectic (tβ) and the average length of the β phase in the eutectic (lβ), μm^2^.

Locations were selected for measurements at 100× magnification, while measurements were performed at 750× magnification to determine the parameters tβ and lβ, and 500× to determine tα.

## 3. Results and Discussion

**Figure 1:**
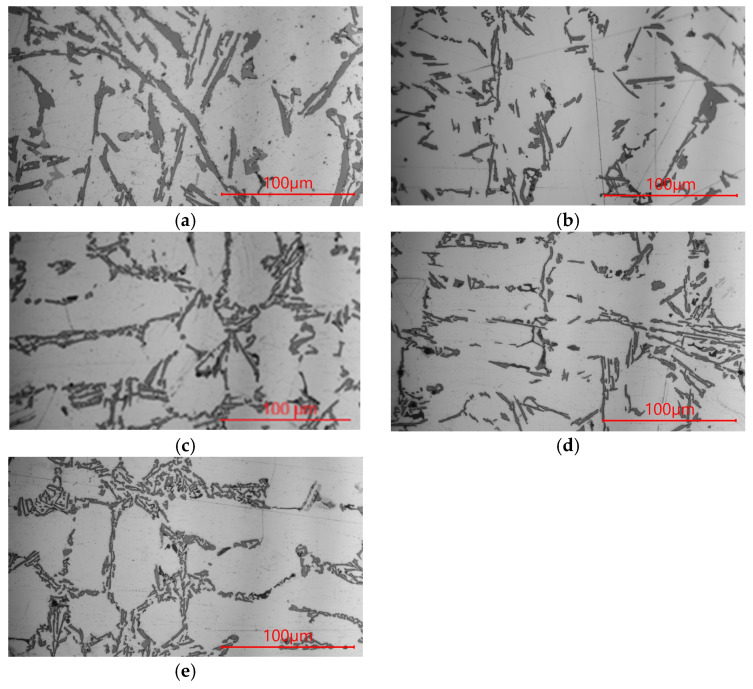
Microstructure of the AlSi9 alloy (**a**) without modifier and with (**b**) Al; (**c**) AlTi; (**d**) AlB; (**e**) AlTiB.

**Figure 2:**
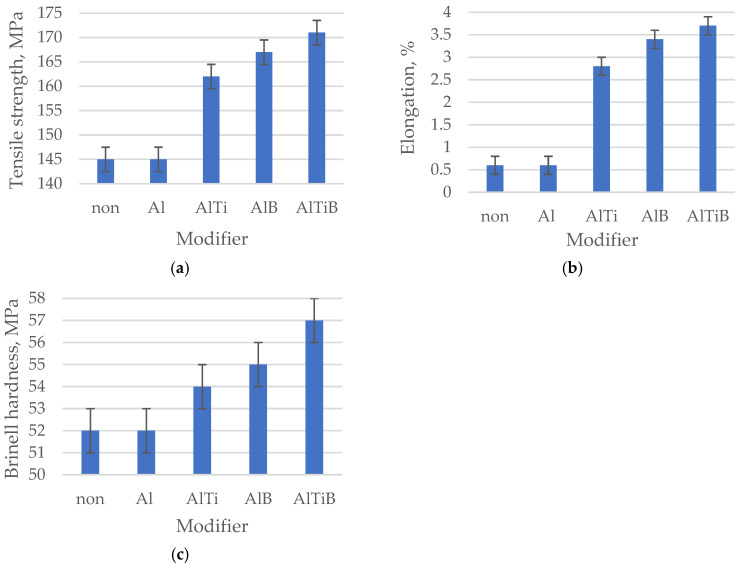
Mechanical properties of the AlSi9M alloy without modifier (non) or with Al, Ti and B: (**a**) Tensile strength. (**b**) Elongations. (**c**) Brinell hardness.

The microstructure of the AlSi9 alloy after processing in the unprocessed state and after processing with the addition of Al, Ti and B is shown in Figure 1, and their mechanical properties are shown in Figure 2.

The stereological parameters of the AlSi9 alloy without treatment and with Al, AlTi, AlB and AlTiB are presented in Table 2.

The AlSi9 alloy in its initial state has thick plates of the eutectic β phase against the background of the large-sized α phase, Figure 1a (tβ = 3.59 μm, lβ = 28.42 μm, aβ = 102.03 μm^2^, Table 2; tα could not be determined due to the inability to isolate dendrites). This microstructure is the reason for the low tensile strength of the silumin of 145 MPa (Figure 2a), elongation A = 0.6% (Figure 2b) and hardness of 52 HB (Figure 2c). After introducing 0.8% Al powder in relation to the mass of the processed silumin into the silumin, a slight refinement of the eutectic β phase was noted. The eutectic (α + β) formed finer regions, Figure 1b (tβ = 2.74 μm, lβ = 24.03 μm, tα = 37.91 μm, aβ = 65.84 μm^2^, Table 2). Changes in the microstructure after aluminum processing were so small that they did not change the mechanical properties of the base silumin (Figure 2a–c). Processing of silumin with an AlTi master alloy containing 0.8% Al and 0.04% Ti based on the mass of the processed alloy resulted in systematization of the eutectic (α + β) and its significant refinement, Figure 1c (tβ = 2.34 μm, lβ = 23.22 μm, tα = 36.56 μm, aβ = 54.34 μm^2^, Table 2). The eutectic β phase plates were partially arranged in parallel, which may indicate partial stabilization of the crystallization process. Changes in the microstructure were reflected in a significant increase in tensile strength to 162 MPa (Figure 2a), elongation A = 2.6% (Figure 2b), and hardness 54 HB (Figure 2c). After processing of silumin with an AlTi master alloy containing 0.8% Al and 0.02% B, thinning of the eutectic β phase plates and a reduction in the dimensions of the original α phase were observed, compared to the AlTi master alloy. The eutectic gained even more distinct parallel platelets, as seen in Figure 1d (tβ = 1.42 μm, lβ = 21.49 μm, tα = 30.64 μm, aβ = 30.52 μm^2^, Table 2). The effects of the above microstructural changes are higher mechanical properties than in the previously described case: tensile strength Rm = 167 MPa (Figure 2a), elongation A = 3.4% (Figure 2b), and hardness 55 HB (Figure 2c). Processing the master alloy containing 0.04% Ti, 0.02% B, and 0.8% Al resulted in further refinement of the microstructure, Figure 1e (tβ = 1.15 μm, lβ = 10.1 μm, tα = 28.66 μm, aβ = 11.96 μm^2^, Table 2). The eutectic β phase is composed of short thin platelets still arranged parallel to the primary α phase. The dimensions of the α phase are similar to the dimensions of this phase after processing the master alloy AlTi (Figure 1c). For this test point, the highest mechanical properties were obtained after using the Al-based master alloy (Figure 2), strength Rm = 171 MPa (Figure 2a), elongation A = 3.7% (Figure 2b) and hardness 57 HB (Figure 2c).

**Figure 3:**
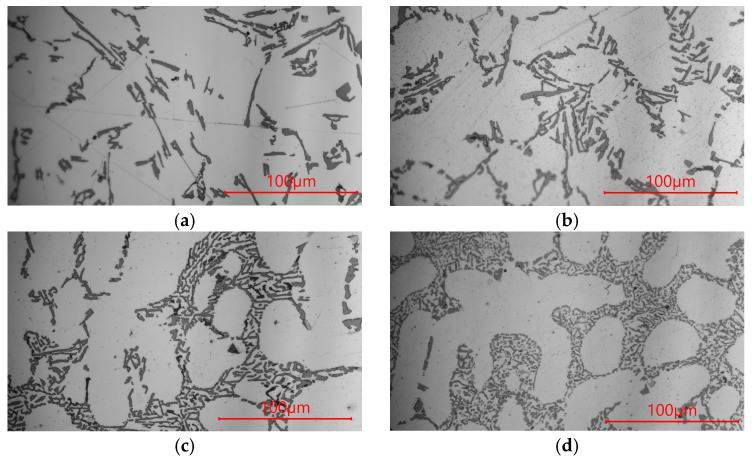
Microstructure the AlSi9 alloy with: (**a**) Al + 7%Si; (**b**) Al + 7%Si + Ti; (**c**) Al + 7%Si + B; (**d**) Al + 7%Si + TiB.

**Figure 4:**
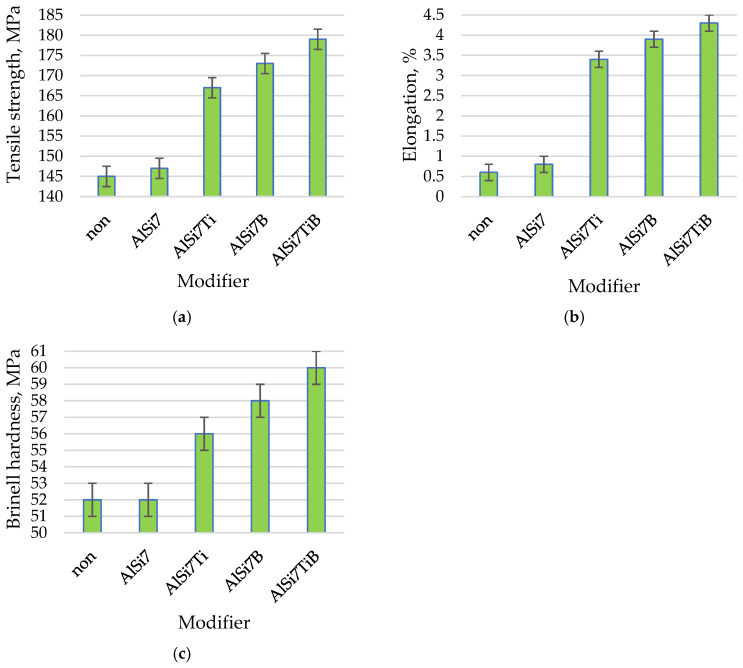
Mechanical properties of the AlSi9 alloy without modifier (non) or with Al + 7%Si + (Ti and B): (**a**) Tensile strength. (**b**) Elongations. (**c**) Brinell hardness.

The microstructures of AlSi9 silumin after treatment of the master alloy based on Al-7% Si with the addition of Ti and B are shown in Figure 3, and the mechanical properties of the alloy are shown in Figure 4. After treatment of the silumin master alloy 0.8% AlSi7, in relation to the alloy cast without additives (Figure 1a), a slight refinement of the eutectic β phase was obtained, as seen in Figure 3a (tβ = 1.90 μm, lβ = 18.01 μm, tα = 31.46 μm, aβ = 34.22 μm^2^, Table 3), but greater than after treatment of 0.8% Al (Figure 1b). However, no significant increase in mechanical properties was noted with respect to the output alloy Rm = 147 MPA, A = 0.8% and H = 52 HB. After introducing 0.04% Ti into the silumin in the master alloy AlSi7, further refinement of the eutectic (α + β) was obtained, as seen in Figure 3b (tβ = 1.34 μm, lβ = 15.53 μm, tα = 33.31 μm, aβ = 20.81 μm^2^, Table 3), to a slightly greater extent than after treatment of the master alloy AlTi (Figure 1c). This refinement resulted in an increase in tensile strength by 22 MPa to 167 MPa (Figure 4a), elongation by 2.8% to 3.4% (Figure 4b), and hardness by 4 HB to 56 HB (Figure 4c). After treatment the master alloy AlSi7 with 0.02% B, the character of the eutectic β phase remained lamellar, while a distinct refinement of the eutectic was observed, as seen in Figure 3c (tβ = 0.92 μm, lβ = 6.24 μm, tα = 25.48 μm, aβ = 5.47 μm^2^, Table 3). This microstructure favored an increase in the mechanical properties of the machined alloy to Rm = 173 MPa, A = 3.9%, and H = 58 HB. The microstructure and mechanical properties after AlSi7 + B treatment showed a higher refinement of the alloy than after AlSi + Ti treatment (Figure 3 and Figure 4). For the master alloy AlSi7 with 0.04% Ti and 0.02% B (relative to the mass of the processed silumin), the finest microstructure was obtained in the conducted tests. It still had a lamellar character; however, the eutectic consisted of a fine β phase, as seen in Figure 3d tβ = 0.81 μm, lβ = 3.73 μm, tα = 21.02 μm, aβ = 3.02 μm^2^, Table 3). At the same time, it was significantly more refined than after processing the master alloy AlTiB (Figure 1e). For this data point, the highest mechanical properties were obtained according to the conducted test plan (Figure 4). The obtained strength Rm = 179 MPa (Figure 4a), elongation A = 4.3% (Figure 4b), and hardness H = 60 HB (Figure 4c). All microstructures obtained after introducing the master alloy AlSi7 (Figure 3) into silumin with titanium or boron additions have a finer eutectic structure (α + β) in relation to aluminum as the base of the master alloy (Figure 1), which translates into obtaining higher mechanical properties for the analogous additions of titanium or boron (Figure 4).

The stereological parameters of the AlSi9 alloy without treatment and with Al + 7%Si, Al + 7%Si + Ti, Al + 7%Si + B, Al + 7%Si + TiB are presented in Table 3.

**Figure 5:**
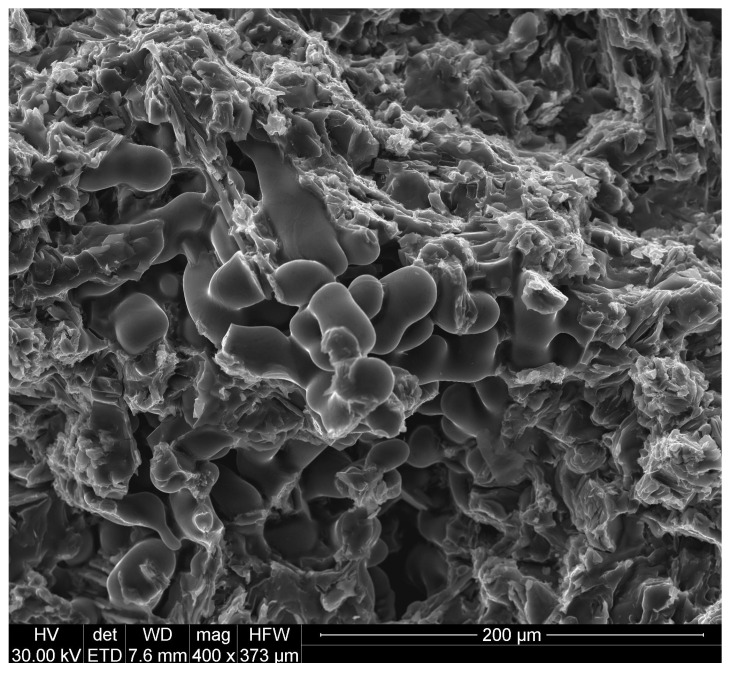
The AlSi9 alloy with Al + 7%Si + TiB.

Figure 5 shows a fracture of an AlSi9 alloy with Al + 7% Si + TiB obtained by fracture of a strength test specimen. It has a mixed, high-energy character with elements of an intergranular fracture. The vast majority of the fractures resulted from energy absorption during plastic deformation. A few areas of material cohesion are visible between the dendrites in the interdendritic spaces. This confirms the relatively high mechanical properties of the alloy.

**Figure 6:**
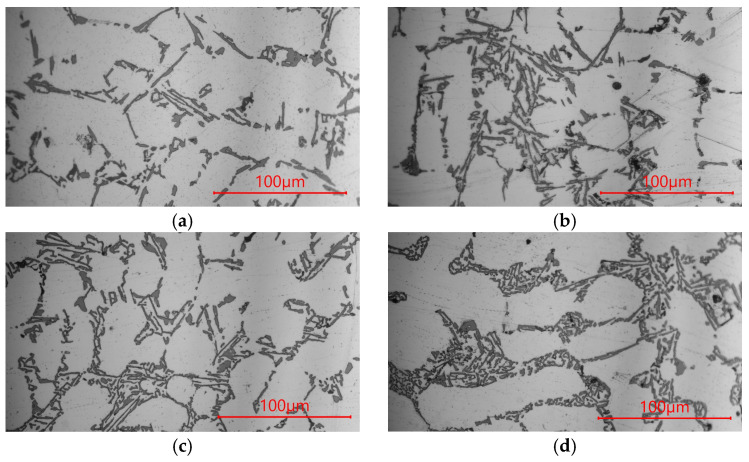
Microstructure the AlSi9 alloy with (**a**) Al + 7%Si + Mg; (**b**) Al + 7%Si + Mg + Ti; (**c**) Al + 7%Si + Mg + B; (**d**) Al + 7%Si + Mg + TiB.

**Figure 7:**
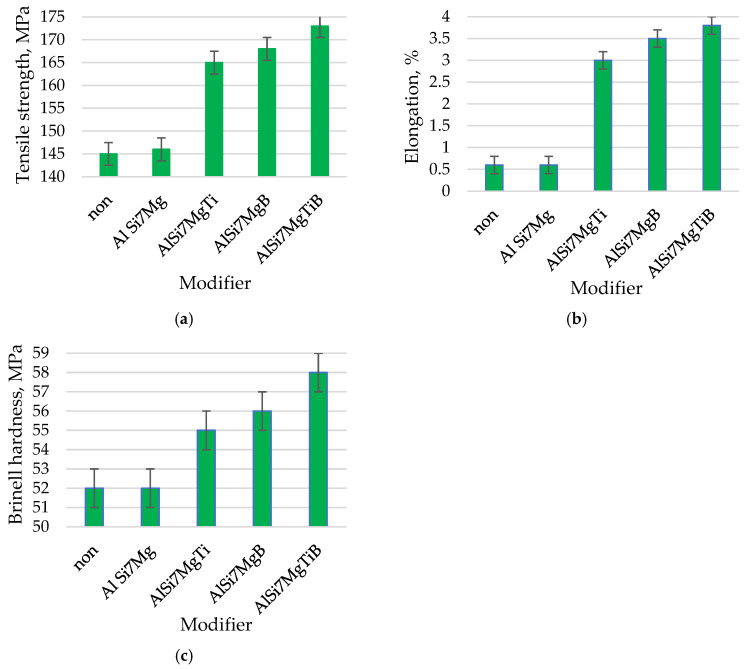
Mechanical properties of the AlSi9Mg alloy without modifier (non) or with Al + 7%Si + Mg + (Ti and B): (**a**) Tensile strength. (**b**) Elongations. (**c**) Brinell hardness.

In the third series of tests, the effect of titanium and boron introduced into a master alloy containing aluminum, silicon, and magnesium was examined (Figure 6 and Figure 7). After introducing 0.8% AlSi7Mg in powder form into the AlSi9 master alloy, no significant visual changes in the microstructure were observed compared to the initial alloy (Figure 6a). Changes were observed in stereoscopic parameters. These consisted of fragmentation of both the eutectic B phase and the dendrite arms of the a phase (tβ = 2.63 μm, lβ = 22.86 μm, tα = 32.37 μm, aβ = 60.12 μm^2^, Table 4). Slightly finer plates of the eutectic β phase were obtained. This microstructure resembles the microstructure after treatment of the Al master alloy (Figure 1a). The mechanical properties after introducing AlSi7Mg did not change with respect to the initial alloy. The introduction of 0.04% Ti into the AlSi7Mg master alloy caused a refinement of the eutectic (α + β), as seen in Figure 6b (tβ = 1.88 μm, lβ = 20.08 μm, tα = 28.92 μm, aβ = 37.75 μm^2^, Table 4), which increased the tensile strength to 165 MPa (Figure 7a), comparable to that after introducing the AlSi7 master alloy (Figure 4a). Elongation increased to 3% (Figure 7b) and hardness to 55 HB (Figure 7c), which constitutes a decrease in elongation by 12% in relation to silumin treated with master alloy AlSi7Ti (Figure 4b). After introducing 0.02% of boron into the master alloy AlSi7Mg, further refinement of the eutectic β phase was noted, as seen in Figure 6c (tβ = 1.26 μm, lβ = 12.79 μm, tα = 27.45 μm, aβ = 16.12 μm^2^, Table 4); however, it is slightly smaller than after introducing boron into the master alloy AlSi7 (Figure 3c). Strength increased to 168 MPa (Figure 7a), elongation to 3.5% and hardness to 56 HB. The obtained values, however, are lower than for the analogous master alloy without Mg addition (Figure 4). The introduction of master alloy AlSi7Mg containing 0.04% Ti and 0.02% B into the silumin was the reason for obtaining a relatively fine eutectic (α + β), Figure 6d (tβ = 0.95 μm, lβ = 6.35 μm, tα = 25.98 μm, aβ = 6.03 μm^2^, Table 4). The microstructure is reflected in the mechanical properties (Figure 7). Tensile strength Rm = 173 MPa (Figure 7a), elongation A = 3.8% (Figure 7b) and hardness H = 58 HB (Figure 7c).

The stereological parameters of the AlSi9 alloy without treatment and with Al + 7%Si + Mg, Al + 7%Si + Mg + Ti, Al + 7%Si + Mg + B and Al + 7%Si + Mg + TiB are presented in Table 4.

**Figure 8:**
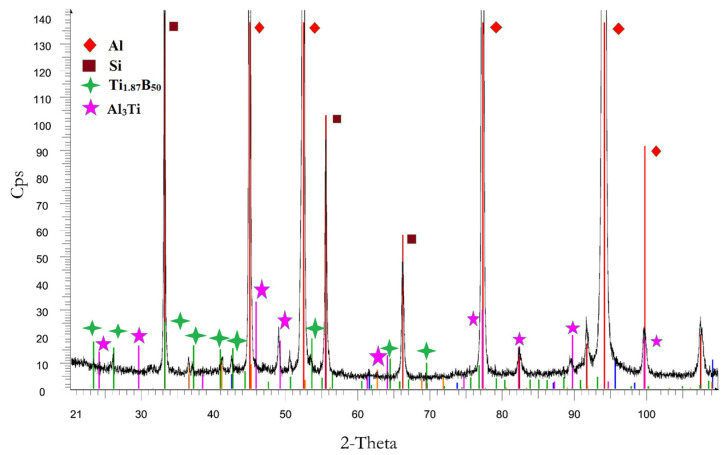
XRD of AlSi9 alloy with AlSi7TiB.

To summarize, this research presented the improvement of hypoeutectic silumin, for example, the AlSi9 alloy, with a master alloy containing titanium and boron, which affects the refinement of the alloy microstructure, which is visible based on microscopic observations (Figure 1, Figure 3 and Figure 5). The sterometric parameters confirm the observations (Table 2, Table 3 and Table 4). XRD analysis (Figure 8) showed the presence of titanium and boron chemical compounds in the alloy, thus confirming the effectiveness of their introduction into the processed silumin. It was found that the additions of titanium or boron to the AlSi7 master alloy containing magnesium refine the microstructure to a lesser extent (Figure 6, Table 4) and increase the analyzed mechanical properties (Figure 7) than for AlSi7 (Figure 3, Table 3 and Figure 4, respectively). However, higher properties were noted for the AlSi7Mg master alloy (Figure 6 and Figure 7) compared to the analogous contents of the components in the Al-based master alloy (Figure 1, Table 2 and Figure 2, respectively). This confirms the results of the work [72] relating the intensity of the interaction of AlB2 with Si. It was noticed that, for analogous shares of titanium and boron, greater microstructure refinement and higher mechanical properties were obtained for the master alloy based on AlSi7 (Figure 4, Table 3 and Figure 5, respectively), slightly lower for the master alloy with magnesium (Figure 6, Table 4 and Figure 7, respectively) and the lowest among the analyzed variants for the master alloy based on aluminum (Figure 1, Table 2 and Figure 3, respectively). The eutectic microstructures of all analyzed samples consisted of lamellar silicon, characteristic of modification with titanium and boron [73,77,78,79,80,81] (Figure 1, Figure 3 and Figure 6). Even for the most improved microstructure among the analyzed points of the research plan, the fracture obtained as a result of stretching the sample has mixed fractography (Figure 5). This indicates further possibilities of research on the improvement of hypoeutectic silumins with a master alloy containing titanium and boron. In all studied variants, the eutectic β-phase plates tend to achieve parallel alignment of their symmetry axes. This demonstrates the tendency toward stabilization and order in the crystallization process. This state is reflected in the mechanical properties represented by tensile strength, elongation, and hardness. All of these properties increased with increasing order (confirmed by the parallel arrangement of the β-phase axes in the eutectic) and with the refinement of the β-phase length.

The average mechanical properties of the AlSi9 alloy with different master alloys are shown in Table 5.

The test results indicate a more intense effect of boron than titanium introduced into the master alloy. However, the simultaneous introduction of both components produces more favorable results both in terms of microstructure refinement and the closely related mechanical properties of silumin. The expectation of the formation of fine-grained Mg_2_Si in the master alloy environment, which, due to the C_1_ crystallographic cell and lattice parameters, could act as a silicon nucleation pad, causing refinement of the eutectic β phase and resulting in increased mechanical properties of the processed alloy, was not confirmed. The microstructure after the introduction of magnesium into the silumin (Figure 6) not only did not undergo refinement but was observed to thicken compared to the same chemical composition of the master alloy, but without magnesium (Figure 3). It is reasonable to assume that the microstructure after the introduction of magnesium resulted in local thickening of the β-phase lamellae, leading to the formation of a granular phase (Figure 6). Such changes in the microstructure are the reason for the decrease in the properties of the alloy machined from the master alloy with magnesium (Figure 7) in relation to the silumin machined from the master alloy without magnesium (Figure 4). For all three test series presented, the greatest microstructure refinement and the resulting highest mechanical properties in relation to the results of each series were obtained after the introduction of the master alloy containing both titanium and boron (Figure 2, Figure 3, Figure 4, Figure 6 and Figure 7).

The presented research results expand the literature on the use of titanium and boron in master alloys to refine the microstructure and enhance the mechanical properties of hypoeutectic silumin. Based on the results of the presented research, it was concluded that the introduction of titanium and boron is most advantageous when performed in a master alloy with a composition similar to that of the alloy being processed. An AlSi7-based master alloy with the addition of 0.04% Ti and 0.02% B can be used to refine the silumin microstructure (primarily electrical) and enhance the alloy’s mechanical properties. It has also been noted that the addition of magnesium in the master alloy reduces the silumin’s mechanical properties.

## 4. Conclusions

Based on the results of the conducted tests, it was concluded that

- It is possible to refine the microstructure using titanium or boron introduced into the master alloys based on Al, AlSi7, and AlSi7Mg;
- The effect of the same amounts of titanium or boron on changes in the microstructure and mechanical properties in the analyzed test plan was highest for the master alloy based on AlSi7;
- The addition of magnesium to AlSi7TiB caused a reduction in the analyzed mechanical properties by several percentages;
- As a result of treatment the aluminum-based master alloy containing titanium and boron, compared to the untreated alloy, the tensile strength increased by 26 MPa (18%), elongation by 3.1% (representing a 516% increase), and Brinell hardness by 5 HB (10%);
- As a result of treatment, the AlSi7-based master alloy containing titanium and boron, compared to the untreated alloy, the tensile strength increased by 34 MPa (23%), elongation by 3.7% (representing a 616% increase), and Brinell hardness by 8 HB (15%). The addition of magnesium, titanium, and boron increased the tensile strength by 28 MPa (19%), elongation by 3.2% (representing a 533% increase), and Brinell hardness by 6 HB (12%).

## Figures and Tables

**Table 1:** Real chemical compositions of testedAlSi9 [103] alloy.

Chemical Element	Si wt. %	Mg wt. %	Mn wt. %	Fe wt. %	Cu wt. %	Ni wt. %	Cr wt. %	Zn wt. %	Na wt. %	Ti wt. %	B wt. %	Al wt. %
Average contents	9.20	<0.01	0.05	0.10	0.03	0.01	0.01	0.04	0.00	<0.01	0.00	bal.

**Table 2:** Stereological parameters of the AlSi9 alloy without and with Al, AlTi, AlB and AlTiB.

Stereological	Master Alloy				
Parameters	non	Al.	AlTi	AlB	AlTiB
tβ, μm	3.59	2.74	2.34	1.42	1.15
lβ, μm	28.42	24.03	23.22	21.49	10.4
tα, μm	-	37.91	36.56	30.64	28.66
aβ, μm^2^	102.0278	65.8422	54.3348	30.5158	11.96

**Table 3:** Stereological parameters of the AlSi9 alloy with Al + 7%Si, Al + 7%Si + Ti, Al + 7%Si + B, and Al + 7%Si + TiB.

Stereological	Master Alloy			
Parameters	AlSi7	AlSi7Ti	AlSi7B	AlSi7TiB
tβ, μm	1.9	1.34	0.92	0.81
lβ, μm	18.01	15.53	6.24	3.73
tα, μm	31.46	30.31	25.48	21.02
aβ, μm^2^	34.219	20.8102	5.7408	3.0213

**Table 4:** Stereological parameters of the AlSi9 alloy with Al + 7%Si + Mg, Al + 7%Si + Mg + Ti, Al + 7%Si + Mg + B and Al + 7%Si + Mg + TiB.

Stereological	Master Alloy			
Parameters	AlSi7Mg	AlSi7MgTi	AlSi7MgB	AlSi7MgTiB
tβ, μm	2.63	1.88	1.26	0.95
lβ, μm	22.86	20.08	12.79	6.35
tα, μm	32.37	28.92	27.45	25.98
aβ, μm^2^	60.12	37.75	16.12	6.03

**Table 5:** Average mechanical properties of AlSi9 alloy with different master alloys.

Master Alloy	Rm	A	HB	Master Alloy	Rm	A	HB	Master Alloy	Rm	A	HB
non	145	0.6	52	non	145	0.6	52	non	145	0.6	52
Al	145	0.6	52	AlSi7	147	0.8	52	Al Si7Mg	146	0.6	52
AlTi	162	2.8	54	AlSi7Ti	167	3.4	56	AlSi7MgTi	165	3	55
AlB	167	3.4	55	AlSi7B	173	3.9	58	AlSi7MgB	168	3.5	56
AlTiB	171	3.7	57	AlSi7TiB	179	4.3	60	AlSi7MgTiB	173	3.8	58

## Data Availability

The original contributions presented in this study are included in the article. Further inquiries can be directed to the corresponding author.
